# Genome-wide identification and comprehensive analysis heat shock transcription factor (Hsf) members in asparagus (*Asparagus officinalis*) at the seeding stage under abiotic stresses

**DOI:** 10.1038/s41598-023-45322-w

**Published:** 2023-10-23

**Authors:** Caihua Li, Yuhuan Li, Zeng Zhou, Yudi Huang, Zunzun Tu, Xin Zhuo, Dingyuan Tian, Yibo Liu, Hongli Di, Ze Lin, Mingxin Shi, Xue He, Haiyu Xu, Yi Zheng, Zhongsheng Mu

**Affiliations:** 1 Jilin Academy of Agricultural Sciences, Changchun, Jilin China; 2 Heilongjiang Bayi Agricultural University, Daqing, Heilongjiang China

**Keywords:** Plant sciences, Evolution

## Abstract

Heat shock transcription factors (Hsf) are pivotal as essential transcription factors. They function as direct transcriptional activators of genes regulated by thermal stress and are closely associated with various abiotic stresses. Asparagus (*Asparagus officinalis*) is a vegetable of considerable economic and nutritional significance, abundant in essential vitamins, minerals, and dietary fiber. Nevertheless, asparagus is sensitive to environmental stresses, and specific abiotic stresses harm its yield and quality. In this context, Hsf members have been discerned through the reference genome, and a comprehensive analysis encompassing physical and chemical attributes, evolutionary aspects, motifs, gene structure, *cis*-acting elements, collinearity, and expression patterns under abiotic stresses has been conducted. The findings identified 18 members, categorized into five distinct subgroups. Members within each subgroup exhibited analogous motifs, gene structures, and *cis*-acting elements. Collinearity analysis unveiled a noteworthy pattern, revealing that Hsf members within asparagus shared one, two, and three pairs with counterparts in Arabidopsis, Oryza sativa, and Glycine max, respectively.Furthermore, members displayed tissue-specific expression during the seedling stage, with roots emerging as viable target tissue. Notably, the expression levels of certain members underwent modification under the influence of abiotic stresses. This study establishes a foundational framework for understanding Hsf members and offers valuable insights into the potential application of molecular breeding in the context of asparagus cultivation.

## Introduction

Heat shock transcription factors (Hsf) constitute a family of pivotal transcription factors primarily responsible for regulating the heat shock response and preserving protein homeostasis within cells^1^. Hsf members exhibit a remarkably conserved structure, with the DNA binding domain (DBD) situated at the N-terminus displaying significant conservation. This DBD employs four reverse equilibria to precisely locate and recognize the β-Fold (comprising β1, β2, β3, and β4) as well as three α-structures (α1, α2, and α3)^2^. In addition to the conserved DNA binding domain located at the N-terminus, the oligomerization domain (OD), nuclear localization signals (NLS), nuclear export signals (NES), and the aromatic large hydrophobic and acid amino residues domain (AHA) at the C-terminus constitute the fundamental structural components of Hsf members^3^. The Hsf members can be categorized into subgroups based on variations in the length of amino acid residues between the DNA binding domain (DBD) and the oligomerization domain (OD)^3^. Hsfs can be activated to exert their functions in response to various stress conditions^4^. Hsf proteins function as molecular chaperones, engaging in vital cellular processes. They play a role in preventing the aggregation of misfolded proteins, rectifying misfolded protein structures, and facilitating the degradation of irreparably damaged proteins under stressful conditions^5^. Hsf members have been reported to participate in various aspects of plant growth and development, particularly emphasizing their involvement in responding to both abiotic and biotic stresses^6^. HSF-HSP pathway was considered as a classical thermal regulatory mechanisms in plants^7^, which heat signaling is transmitted through reactive oxygen and calcium ions^8^. *HSFA1* as a regulator of the HSF-HSP pathway, which activates the expression of downstream HSP genes and interacts with HSF members such as *HSFA1* and *HSFB1*. Also, *HSFA1* induces the expression of *DREB2A* directly, which *DREB2A* is a related osmotic and heat stress and positively controls osmotic- and heat-inducible gene and regulates the expression of the *HSFA3* to enhance tolerance of plants^9,10^; In Arabidopsis, *HSFA1b* has been documented to play a role in enhancing plant resistance against *Pseudomonas aeruginosa*. Notably, the fc/a mutant displays heightened sensitivity to this pathogen^11^. In tomato* (Solanum lycopersicum),* the Hsf member known as *SIHSFAla* plays a crucial role in generating extracellular H_2_O_2_ upon nematode infection. This process further facilitates the accumulation of heat shock proteins and augments the foundational defense mechanisms^12^; HSFA4a's function is closely intertwined with cell death processes during pathogen infections. It upregulates the expression of pathogen-responsive genes, contributing to the intricate interplay between plants and pathogens^13^; *HSFB4d,* a member of the Hsf family in rice, is pivotal in enhancing resistance against bacterial wilt disease. It achieves this by inducing the expression of various genes involved in the plant's defense mechanisms^14^.

Additionally, Hsf members have been reported to play a significant role in responding to abiotic stresses^6^: *HsfA1* is an important Hsf member in Arabidopsis known for its role in regulating heat tolerance. Mutants of *HsfA1*, such as hsfa1abcd mutants, show severe impairment in responding to heat stress^15^; Overexpression of *HSFA2* has been shown to restore the heat-sensitive phenotype observed in hsfa1abcd mutants^16^; ABA has been demonstrated to induce the expression of several HSF members, including *HSFA6a*, *HSFA6b*, *HSFA8*, *HSFB2a*, *HSFB2b*, and *HSFC1*^17^; Cold stress can cause the expression of *HSFA4a*, *HSFA4b*, *HSFA8*, and *HSFC1*. Additionally, overexpression of *HSFA1*, *HSFA2*, *HSFA3*, and *HSFAa* has been demonstrated to enhance heat tolerance in plants and improve tolerance to various other stresses, including drought, salt, hypoxia, and osmotic stress^18–21^.

Hsf members have been identified in various plant species, including Arabidopsis^4^, rice (*Oryza sativa*)^22^, maize (*Zea Mays*)^23^, soybean (*Glycine max*)^24^, wheat (*Triticum aestivum*)^25^ and common bean (*Phaseolus vulgaris*)^26^. However, comprehensive research on Hsf members in asparagus (Asparagus officinalis) is lacking. Asparagus is a vegetable of significant nutritional and health value, primarily harvested for its tender stems, which are rich in essential vitamins, minerals, and medicinal properties^27^. Asparagus is known for its abundant nutrients, including vitamins, carbohydrates, proteins, diverse amino acids, and various bioactive compounds such as polyphenols, flavonoids, saponins, and asparaginic acid^28^. These compounds contribute to different dietary effects, such as immune regulation, moistening of the lungs and relieving cough, cardiovascular health support, and potential anti-tumor properties^27^. Renowned as the “king of vegetables” in the global market, asparagus holds a cherished position among consumers worldwide^29^. Nevertheless, asparagus stands vulnerable to various environmental stressors, particularly abiotic stresses, leading to substantial reductions in both yield and quality.

Consequently, enhancing the abiotic stress tolerance of asparagus through molecular breeding has become imperative. In this context, a thorough genome-wide exploration of Heat shock transcription factor (Hsf) members within the reference genome was undertaken. This comprehensive study encompassed an exhaustive analysis of these Hsf members, encompassing their physical and chemical attributes, evolutionary patterns, underlying motifs, gene structural compositions, *cis*-acting elements, collinearity, and expression profiles in response to abiotic stressors. The outcomes of this investigation not only unraveled the unique attributes of Hsf members but also furnished a vital theoretical foundation for the practical application of molecular breeding strategies in asparagus improvement.

## Materials and methods

### The identification of Hsf members in asparagus

Genomic data, encompassing gene, cDNA, and protein information, were sourced from the National Center for Biotechnology Information (NCBI) database (https://www.ncbi.nlm.nih.gov/), with the accession number PRJNA376608 (https://www.ncbi.nlm.nih.gov/genome/10978). The identification of Heat shock transcription factor (Hsf) members was facilitated by utilizing HMMER software, which focused on detecting the domain (PF00447). This approach enabled the comprehensive identification and subsequent exploration of Hsf members within the provided genomic dataset ^30^ while 1e^−15^ was set as a filter threshold. Also, the Hsf members in *Arabidopsis* and rice (*Oryza sativa*) were came from the reference of Busch’s^31^ and Guo’s^22^ results for comprehensive analysis; The roster of identified Hsf members was subjected to a screening process facilitated by ExPASy Proteomics Server and Plant Protein Phosphorylation Database software. This meticulous scrutiny allowed for a more refined selection and analysis of the Hsf members within the dataset^32^ by default parameters. All of the HSF members identified in asparagus after screened was used for subsequent analysis. The selected candidates, resulting from the screening process, were definitively identified as members of the Hsf family, marking the commencement of subsequent in-depth analysis.

### The analysis of Hsf members in asparagus

Various databases, including NCBI and Phytozome, furnished essential details regarding Hsf members, encompassing attributes such as location and coding sequence length, enabling a thorough examination of these members' physical and chemical properties. In parallel, the evolutionary relationship among Hsf members was elucidated through MEGA X software. The optimal model for this analysis was predicted using MEGA X^30^ while while maximum likelihood method and 1000 bootstrap was used in this study, which the suitable model was predicted by MEGA X. Moreover, the reference sources for Hsf members in *Arabidopsis* and rice (*Oryza sativa*) were derived from Busch's work^31^ and Guo’s^33^ results. For motif analysis of Hsf members, the MEME software was harnessed, with the criterion that each motif's value was below 1e^−20^. Indeed, the length of each motif encompassed a range of 10 to 50 amino acids^34^. Indeed, the Gene Structure Display Server (GSDS) software was employed to analyze the gene structure of the Hsf members. Additionally, the Gene-wise software facilitated the analysis of DNA coordinates, including exonic and intronic regions, as well as coding sequence and protein sequences^35^, which filter according to the default parameters of the software; Indeed, the PlantCARE software was utilized to predict the *cis*-acting elements of the Hsf members, providing a comprehensive analysis of these elements^36^, which 0.01 was set as a filter default parameters; The collinear relationship of the Hsf members was analyzed using the Circos and MCScanX software tools^37^, which the location information and cds sequences of two species' members, and filtering the collinear regions, all collinear gene pairs are ultimately obtained by McScanX.

### The expression pattern analysis of Hsf members in asparagus

The expression patterns of Hsf members in asparagus were studied using Jinglvlu III, a locally grown variety provided by the Jilin Academy of Agricultural Sciences. Four-month-old seedlings with normal growth were selected as the plant materials. These seedlings were divided into different tissues, including roots, stems, and leaves, for the analysis of tissue-specific expression using the quantitative real-time PCR (qRT-PCR) method^38^. Subsequently, the roots were chosen as the target tissue to analyze the expression of Hsf members under abiotic stresses. This analysis designated water treatment as the control (CK) treatment. At the same time, 70 mmol/L NaCl was employed as the salt stress (S) treatment^39^, A temperature of 43 °C was designated as the heat stress (H) treatment, and a temperature of 4 °C was selected as the cold stress (C) treatment^39^; After subjecting the samples to stress treatments for 48 h, the target tissues were collected for subsequent expression pattern analysis. The RNA from each sample was extracted using the MolPure® RNA Kit (19,291, YEASEN, Shanghai, China). The extracted RNA was then reverse transcribed into cDNA using the Evo M-MLV reverse transcription reagent premix (AG11706, AG, Hunan) after its quality was assessed by 1% agarose gel electrophoresis and a NanoDrop instrument (OneC, Thermo Fisher, Waltham, MA, USA).

To determine the expression levels of Hsf members, qRT-PCR analysis was conducted. The primers for amplifying Hsf members were designed, and 2 × SYBR qPCR Master Mix (M4QS02, bioeast, Hangzhou, China) was utilized. The LightCycler® system (Roche 480II, Roche, Switzerland) was employed to analyze the expression levels of Hsf members, and the experiments were carried out with three biological replicates. The relative expression level was calculated using the 2^−ΔΔCt^ method^40^.

### Statement

The plant materials used in this experiment are local cultivated varieties, which comply with national guidelines and legislation. All the methods were carried out in accordance with relevant Institutional guidelines and regulations.

## Results

### The identification of Hsf members in asparagus (AoHsfs)

**Figure 1:**
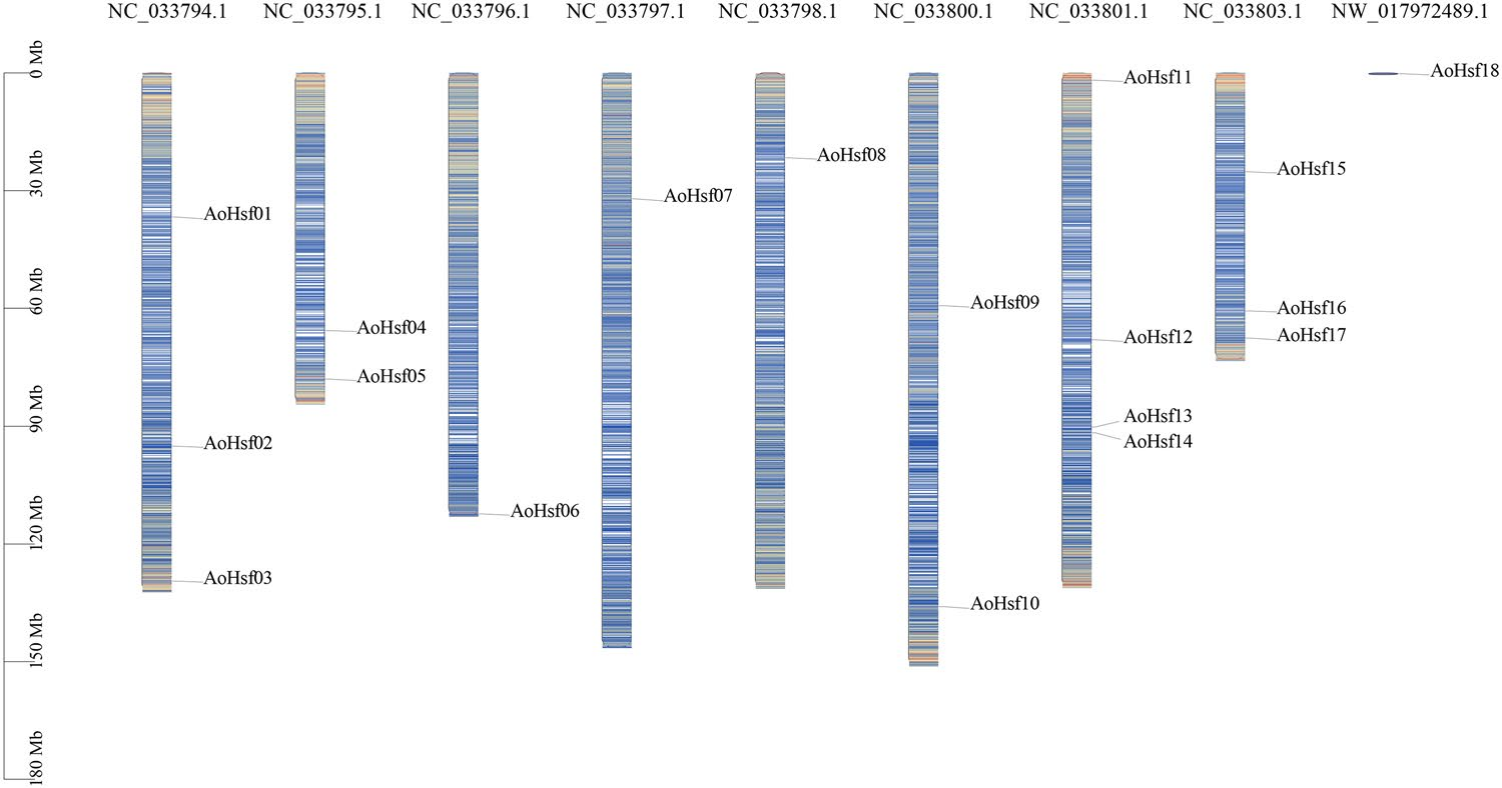
The spatial distribution of AoHsf members was illustrated in the analysis. The vertical number axis on the left represented the chromosomal ruler, indicating the positions of chromosomes. The members were depicted by black lines, signifying their locations along the chromosomes. To denote gene density on the chromosomes, the lines were colored differently. The color gradient ranged from red to blue, symbolizing the gene density from high to low respectively.

In this study, 21 members were initially identified through the reference genome. However, three redundant members were found after removing the duplicates, leaving a final count of 18 unique members. These members were then designated with names based on their chromosomal arrangement, resulting in the nomenclature AoHsf01 to AoHsf08, respectively. The analysis revealed that these AoHsf members were distributed across eight different chromosomes. Notably, AoHsf08 was not associated with any specific chromosome (NW_017972489.1). Among these, four members, namely AoHsf11, AoHsf12, AoHsf13, and AoHsf14, were located on chromosome NC_033803.1 (Fig. 1).

Detailed information regarding the AoHsf members was retrieved from the NCBI and Phytozome databases and is provided in Table S2. The length of the AoHsf proteins ranged from 170 to 504 amino acids. Specifically, AoHsf02 exhibited the largest protein length, while *AoHsf06* had the shortest. The instability index of the AoHsf members varied between 42.04 (*AoHsf02*) and 67.62 (*AoHsf06*). Furthermore, the aliphatic index of these members ranged from 54.61 to 80.96. The comprehensive information compiled here serves as a foundational resource for understanding the characteristics of the AoHsf members.

### The evolution of AoHsfs

**Figure 2:**
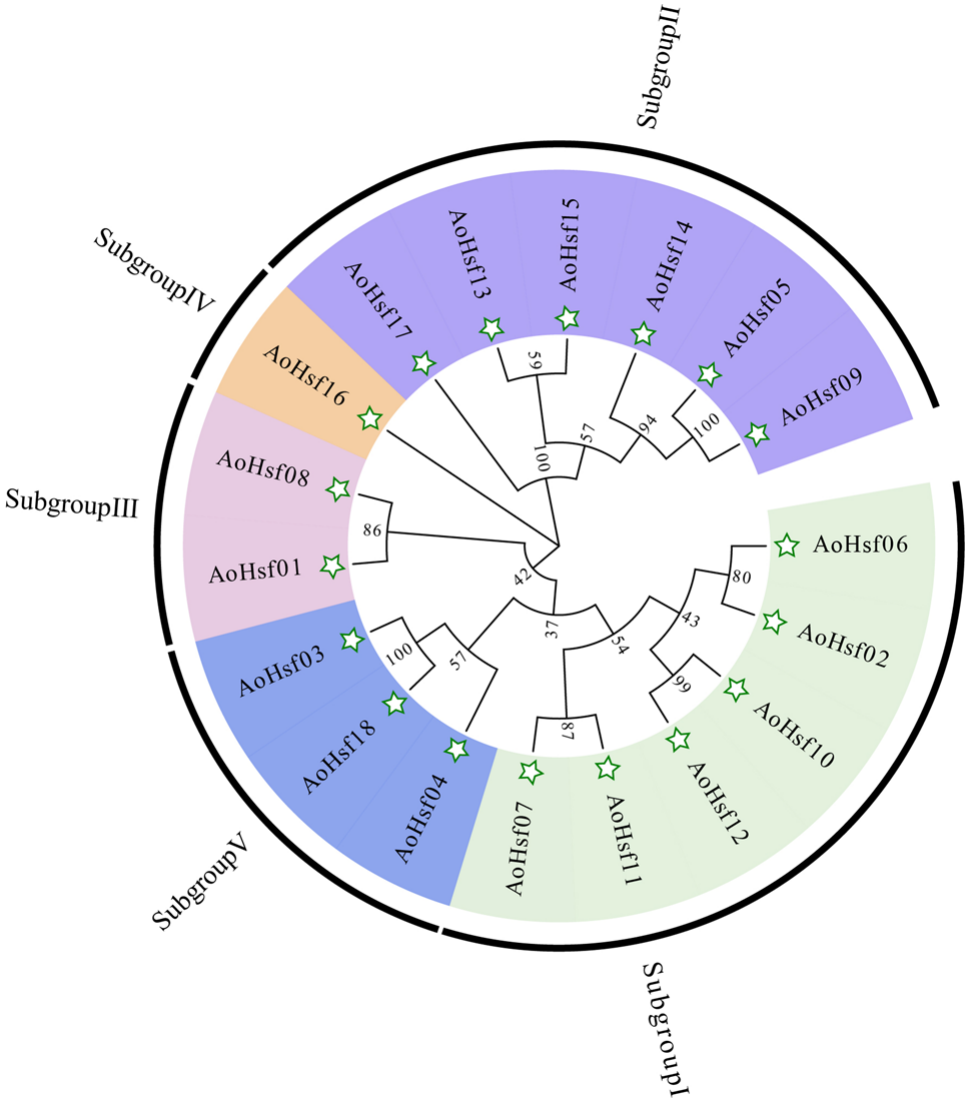
The evolutionary relationship of AoHsfs. The green stars were used to indicate AoHsf members and the background was color-coded, with green, purple, pink, orange, and blue representing subgroup I, II, III, IV, and V, respectively. The numbers displayed on the tree corresponded to the evolutionary values of the AoHsfs.

The protein sequences of AoHsfs were subjected to detection and analysis using MEGA X software. For optimization, the jtt+g+i model was selected based on predictions by MEGA X. The outcome revealed the classification of the 18 AoHsf members into five distinct clusters, designated as subgroup I, II, III, IV, and V (Fig. 2). Among these clusters, subgroup IV contained only one AoHsf member, representing the subgroup with the fewest members. In contrast, subgroup I and II comprised six AoHsf members each, signifying the subgroups with the highest number of members in this classification.

### The motifs and gene structure of AoHsfs

**Figure 3:**
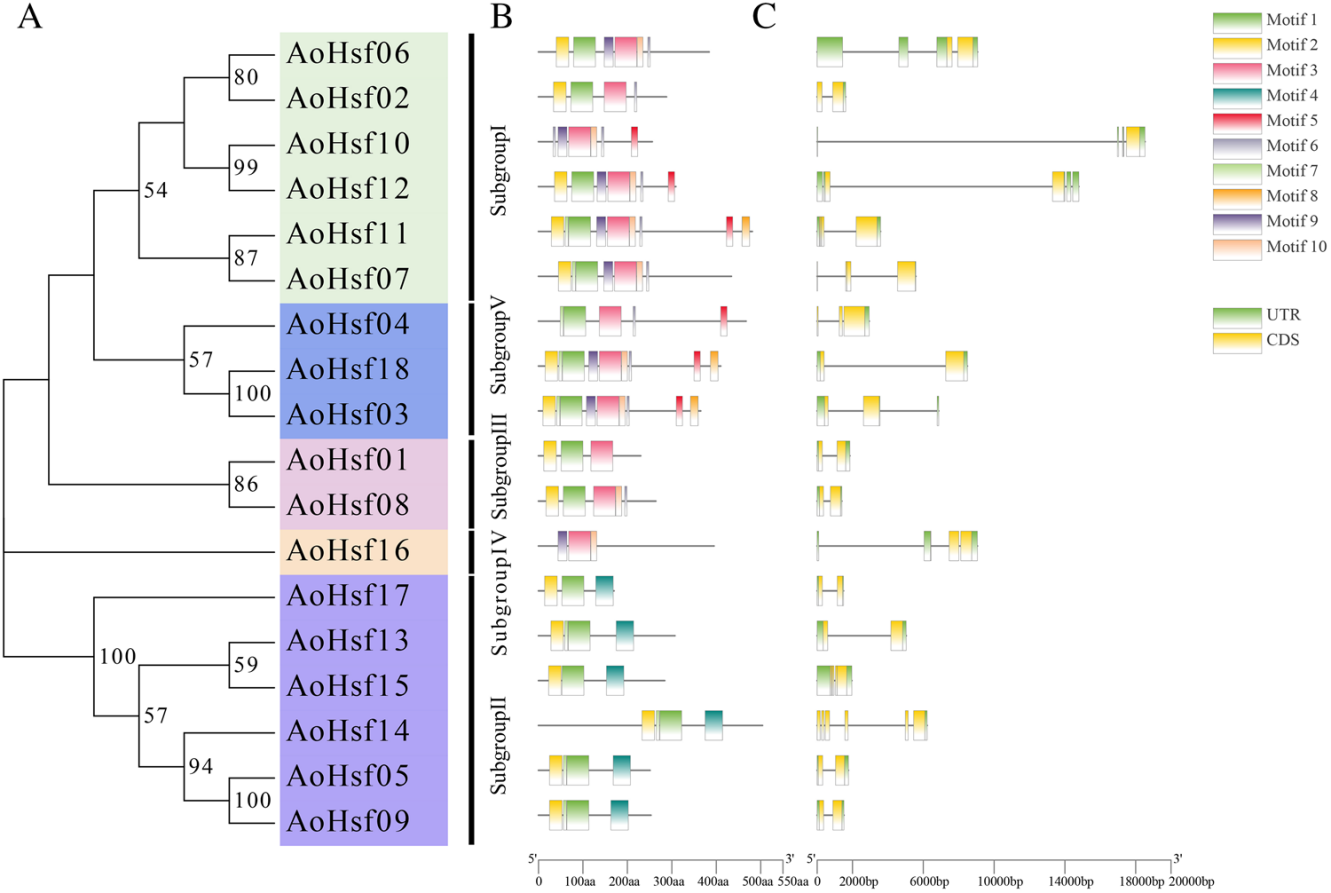
The motifs and gene structure of AoHsfs were intricately analyzed. (**A**) The evolutionary trajectory of AoHsfs, where the five distinct colors signify subgroups I through V. (**B**) Motif representation of AoHsfs. The ten differently colored squares denote the distinct motifs, numbered 1 through 10. (**C**) Illustrates the gene structure of AoHsfs. Here, the green boxes depict the untranslated region (UTR), the yellow boxes indicate the coding sequence (CDS) region and the black lines represent the intron regions.

The motifs and gene structure analysis of AoHsfs was conducted using MEME and GSDS software (Fig. 3). The findings revealed that members within the same subgroup shared similar motifs (Fig. 3A,B and Table S3). Specifically, only subgroup IV lacked motif 1, and subgroup II lacked motifs 3, 5, 6, and 8. Additionally, motif four was exclusively identified in subgroup II. The gene structure analysis results indicated that AoHsfs within the same subgroup also exhibited a similar gene structure. Notably, the longest member was identified in subgroup I, while members in subgroup III were consistently shorter than those in other subgroups (Fig. 3A,C).

### The evolution and motifs of Hsf members

**Figure 4:**
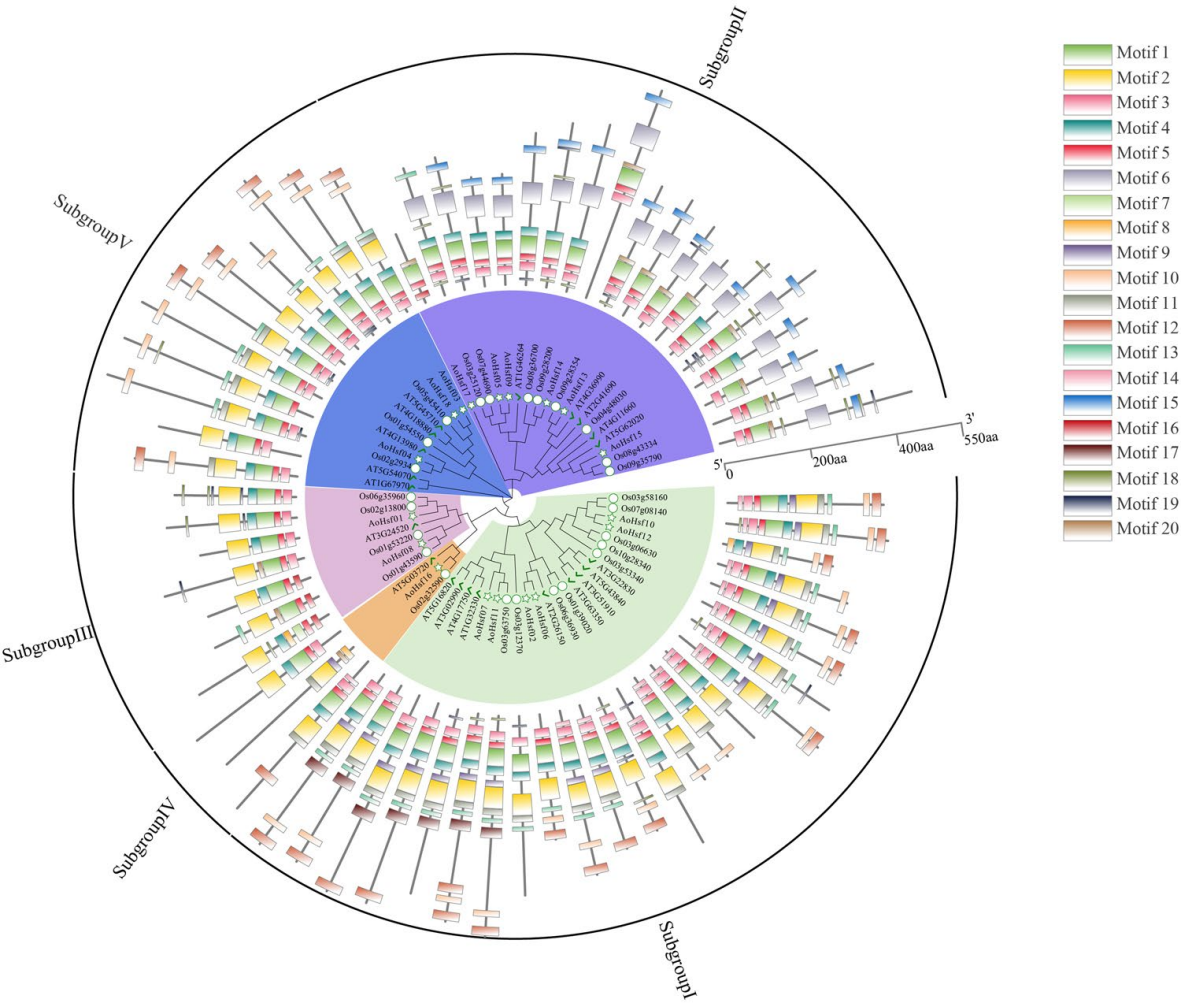
The evolutionary trajectory and motif patterns of Hsf members from *Arabidopsis*, rice (*Oryza sativa*), and asparagus (*Asparagus officinalis*) were extensively analyzed. The inner circle delineates the evolutionary relationships among the Hsf members, while the outer circle showcases the motif distribution across these members. Each of the 20 differently colored boxes signifies a unique motif, ranging from motif 1 to motif 20.

This comprehensive study examined Hsf members across three plant species: Arabidopsis, *Oryza sativa*, and *Asparagus officinalis*. A total of 64 Hsf members were identified from the reference genomes of these species. The evolutionary relationship analysis revealed their classification into five subgroups, with subgroup IV having the lowest number (3 members) and subgroup I containing the most significant number of Hsf members. In the motif analysis, members within the same subgroup displayed similar motif profiles, with specific motifs, such as motif 6 and 15, unique to subgroup II (Fig. 4 and Table S4).

### The *cis*-acting elements of AoHsfs

**Figure 5:**
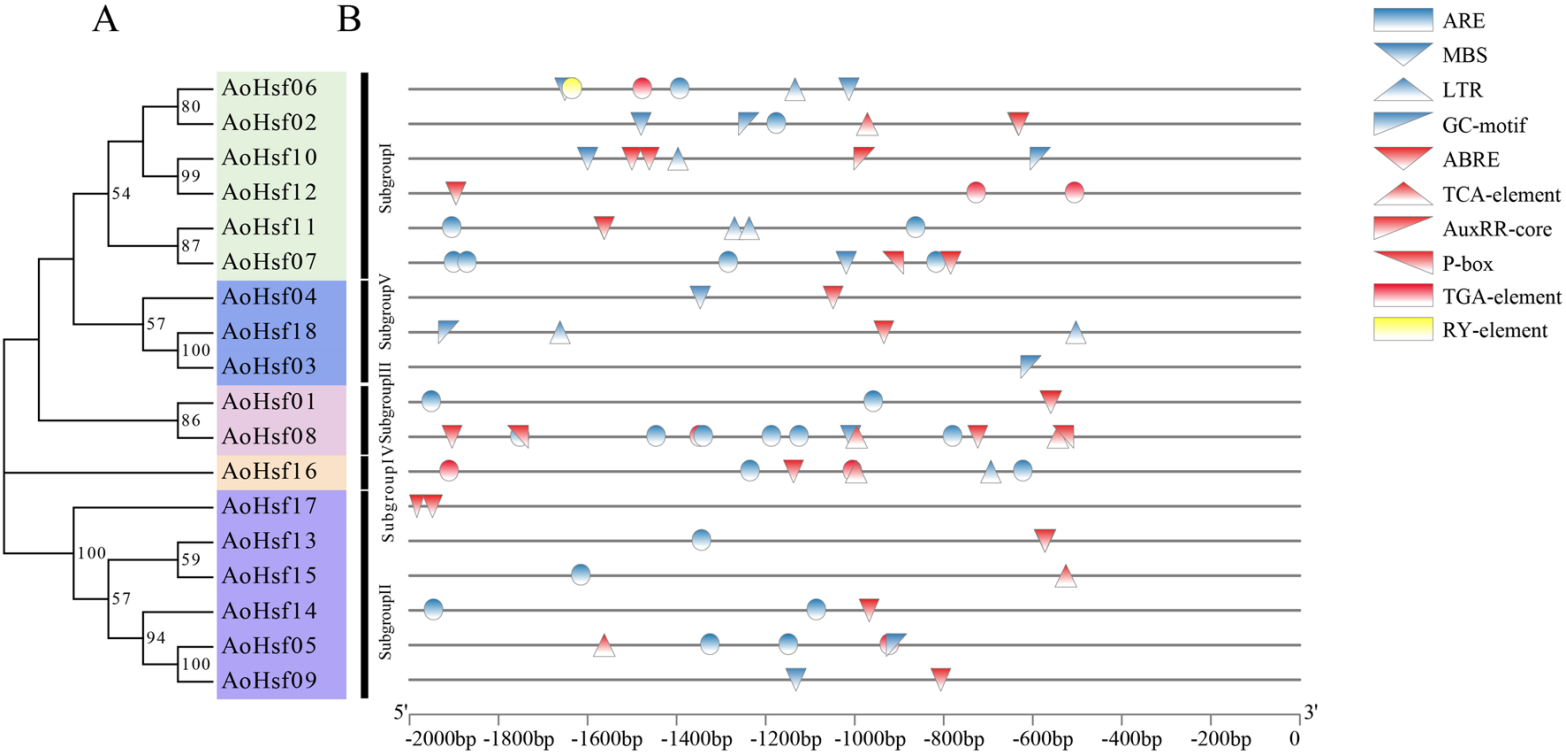
The *cis*-acting elements analysis of AoHsfs. (**A**) The evolution of AoHsfs. Five different colors represented subgroup I–V; (**B**) The *cis*-acting elements of AoHsfs. Blue boxes were the stress-related elements; red boxes were hormone-related elements; Yellow boxes were the elements with sprout-related functions.

In this analysis, the *cis*-acting elements of AoHsfs were thoroughly examined using PlantCare software. The predicted functions of these *cis*-acting elements are detailed in Table 1. These elements were categorized into three types and visualized with different colors in Fig. 5: stress-related elements (blue), hormone-related elements (red), and sprout-related elements (yellow). Nearly all *AoHsf* members possessed hormone-related and stress-related characteristics, except *AoHsf03* (lacking hormone-related elements) and AoHsf17 (lacking stress-related aspects). This analysis underscores the close association between AoHsfs and stress, hormones, and sprouting, revealing their potential roles in these processes. The analysis of *cis*-acting elements in *AoHsfs* revealed three distinct types, represented by different colors in Fig. 5: stress-related elements (blue), hormone-related elements (red), and sprout-related elements (yellow). Almost all *AoHsf* members were found to have both hormone-related and stress-related features, except for *AoHsf03* and *AoHsf17*. This observation highlights the close association between *AoHsfs* and hormonal responses and their involvement in stress-related processes. Notably, *AoHsf06* was identified to contain an RY element, suggesting its potential role during sprouting. These findings underscore the multifaceted participation of *AoHsfs* in stress, hormonal regulation, and sprouting processes.

**Table 1:** The prediction of *cis*-acting elements of AoHsfs.

Element name	The function of *cis*-elements
ARE	*Cis*-acting regulatory element essential for the anaerobic induction
LTR	*Cis*-acting element involved in low-temperature responsiveness
ABRE	*Cis*-acting element involved in the abscisic acid responsiveness
TGA-element	Auxin-responsive element
TCA-element	*Cis*-acting element involved in salicylic acid responsiveness
GC-motif	Enhancer-like element involved in anoxic specific inducibility
MBS	MYB binding site involved in drought-inducibility
RY-element	*Cis*-acting regulatory element involved in seed-specific regulation
P-box	Gibberellin-responsive element
AuxRR-core	*Cis*-acting regulatory element involved in auxin responsiveness

### The collinearity of AoHsfs

**Figure 6:**
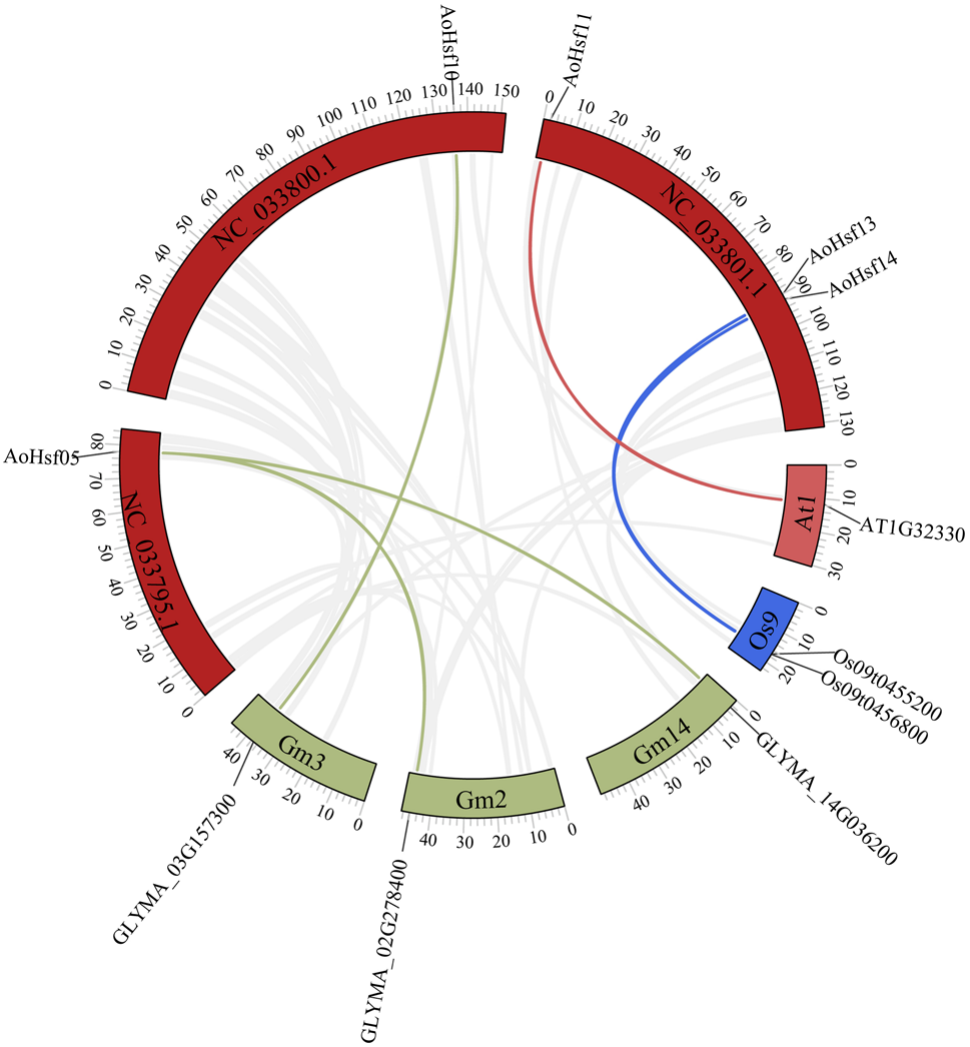
The collinearity analysis of AoHsfs. The red squares represented the chromosomes of asparagus (*Asparagus officinalis*), while the green squares represented the chromosomes of soybean (*Glycine max*); The blue squares represented the chromosomes of rice (*Oryza sativa*), while pink squares represented the chromosomes of Arabidopsis. The bright lines were the collinear relationship with members, while the gray lines were the collinearity background.

Also, the collinear relationship was analyzed. The result of collinearity showed that there were three pairs of collinear genes with soybean (*Glycine max*), which were *AoHsf10* with *GLYMA_03G175300*, *AoHsf05* with *GLYMA_02G278400*, and *AoHsf14* with *GLYMA_02G278400*. Two pairs of collinear genes with rice (*Oryza sativa*) and only one pair had a collinear relationship, *AoHsf11* with *AT1G32330*. All these results revealed that these members might have a close relationship with these collinear genes (Fig. 6 and Table S5).

### The expression patterns of AoHsfs

**Figure 7:**
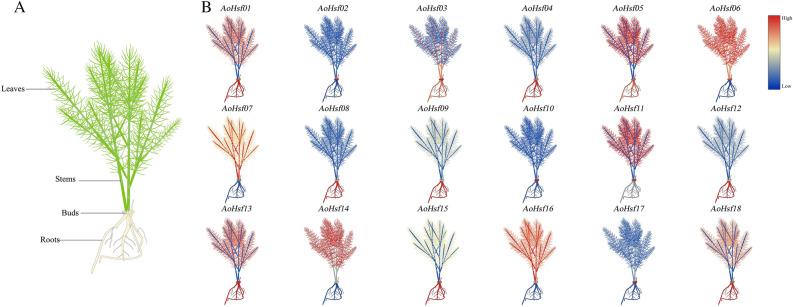
Analysis of AoHsfs' Expression Patterns at the Seeding Stage. (**A**) Schematic representation of seedlings in various tissues, including leaves, stems, buds, and roots; (**B**) Expression patterns of *AoHsfs* across different tissues. Colors range from blue to red, indicating expression levels from low to high.

The expression patterns of AoHsfs in different tissues at the seeding stage were tested by qRT-PCR analysis. The plant was divided into four tissues: leaves, stems, buds, and roots (Fig. 7A). The results showed that almost all AoHsfs had higher expression levels in roots (10) than in leaves, stems, and buds, which suggests that roots might be a target tissue for studying AoHsfs at the seeding stage (Fig. 7B).

### The expression level under abiotic stresses

**Figure 8:**
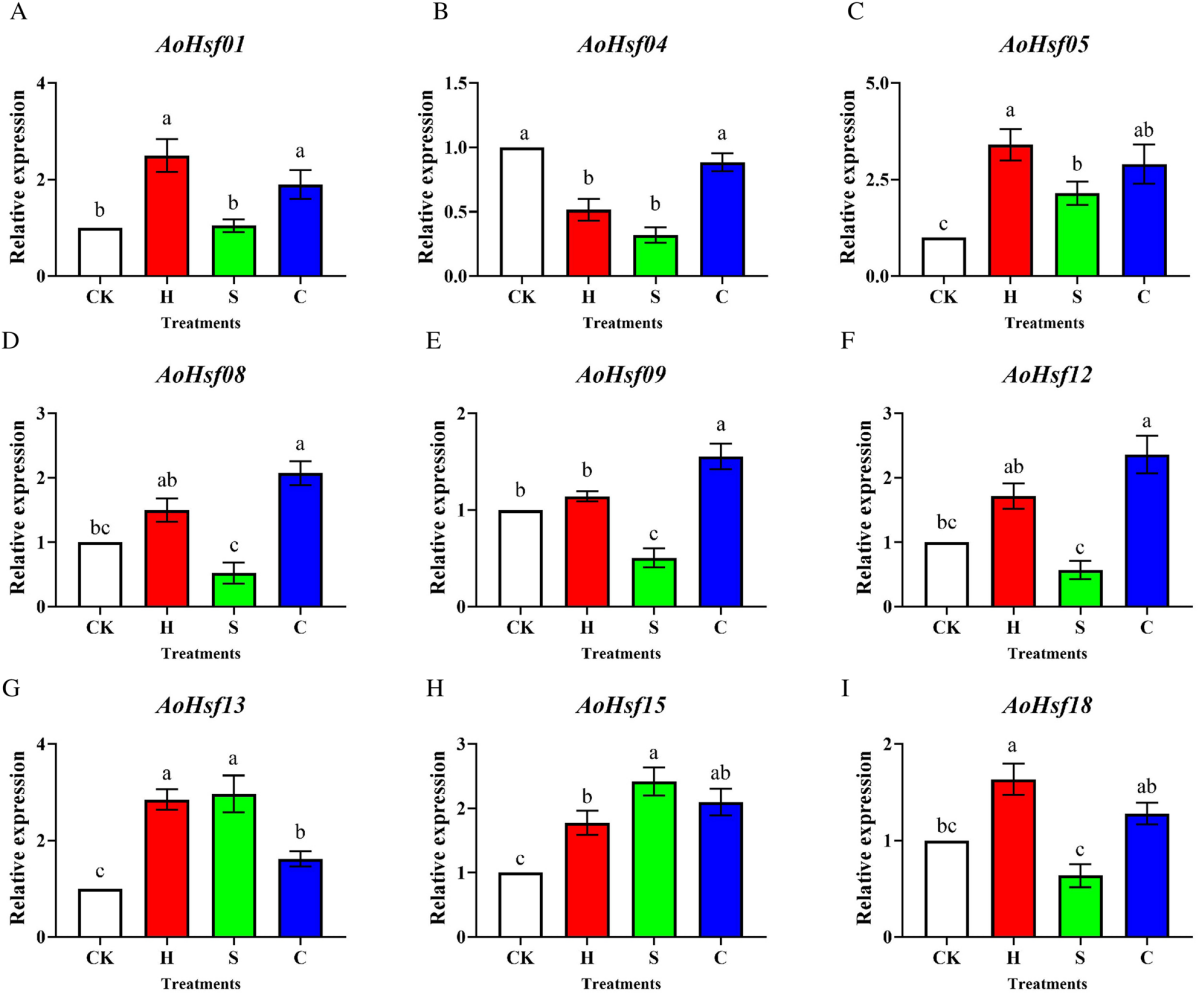
The expression patterns analysis of *AoHsfs* under abiotic stresses. The white column represented CK treatment, the red column represented heat stress treatment, the green column represented salt stress treatment, and the blue column described cold stress treatment. Lowercase letter(s) indicated significant differences (*α* = 0.05). (**A**) The expression of *AoHsf01* exhibited variations across different abiotic stress treatments; (**B**) The expression level of *AoHsf04* under abiotic stresses; (**C**) The expression level of *AoHsf05* under abiotic stresses; (**D**) The expression level of *AoHsf08* under abiotic stresses; (**E**) The expression level of *AoHsf09* under abiotic stresses; (**F**) The expression level of *AoHsf12* under abiotic stresses; (**G**) The expression level of *AoHsf13* under abiotic stresses; (**H**) The expression level of *AoHsf15* under abiotic stresses; (**I**) The expression level of *AoHsf18* under abiotic stresses.

Nine *AoHsfs*, which exhibited higher expression levels in roots, were selected as target genes to investigate their expression changes under abiotic stresses. The results showed that nearly all of these members exhibited increased expression levels under abiotic stresses, including heat, cold, and salt, except for *AoHsf12*, which displayed differential expression patterns under different stresses (Fig. 8). Notably, *AoHsf05*, *AoHsf13*, and *AoHsf15* showed significantly higher expression levels under all three abiotic stress conditions than the control (CK) treatment. These genes hold promise as candidate *AoHsfs* for breeding programs to enhance stress tolerance. Interestingly, most of the AoHSF members with significant changes in differential expression levels had the stress-related elements while the expression of *AoHsf12* had no such great change under stress (Figs. 5, 8), these information also indirectly revealed the potential features of AoHSF members in response to abiotic stresses.

## Discussion

Hsf members have been identified in different species, and the count of Hsf members varies between species. For example, Arabidopsis possesses 21 Hsf members^3^; however, tomato (*Solanum lycopersicum*) has 18 identified Hsf members^4^. From the alfalfa (Medicago sativa) genome, 16 Hsf members were successfully identified^41^ while 30 members were found in common bean (*Phaseolus vulgaris*)^26^; 29 members were identified in buckwheat (*Fagopyrum tataricum*)^42^ Poplar (Populus) revealed a total of 28 Hsf members within its genome^41^. In this study, we identified 18 Hsf members in asparagus through the reference genome in the database. The relatively lower number of Hsf members in asparagus compared to some other species can be attributed to two main factors. First, the reference genome of asparagus is relatively tiny compared to other species. Second, the lower number could result from gene duplication events during genomic replication and evolution^43^.

Furthermore, the Hsf members exhibited diverse classifications in their evolutionary relationships, ranging from 3 to 9 subgroups. This diversity reflects the complexity and differentiation within the Hsf gene family in asparagus^44–46^. In this study, these 18 members were classified into five subgroups, which aligns with the number of subgroups observed in the evolutionary analysis. The evolutionary and motif analyses involving three species (*Arabidopsis*, *Oryza sativa*, *and Asparagus officinalis*) revealed that these 64 members were grouped into five subgroups. Remarkably, each subgroup contained Hsf members from *Arabidopsis*, *Oryza sativa*, *and Asparagus officinalis*. This observation suggests that Hsf members are conserved across monocotyledonous and dicotyledonous plants throughout evolution.

Each subfamily of Hsf members exhibited distinct characteristics based on motifs, gene structure, and *cis*-acting elements. These characteristics were similar to those of Hsf members in various other plant species^46^. Motifs 1 and 2 contained the DBD domain and were present in all *AoHsf* members. Furthermore, members within the same subfamily shared similar motifs. It's worth noting that Hsf members have also been identified in bamboo (*Phyllostachys edulis*)^47^. Similar results have been observed in tea^48^ (*Camellia sinensis*) and maize^23^ (*Zea Mays*). Gene structures, including coding sequences (CDS) and introns, play crucial roles in regulating gene expression levels and can provide insights into gene functions^49^.

The gene structure analysis of *AoHsfs* indicated that members within each subgroup had a similar structure, suggesting potential functional similarities among members within the same subgroup. Similar findings have been reported in common bean and cotton as well^46^; *Cis*-elements located in the promoter regions of genes play a critical role in regulating gene expression levels, which can significantly impact the secondary metabolism of plants and influence various aspects of their biology^36^. The analysis of *cis*-acting elements in *AoHsf* members revealed the presence of different hormone-related elements (including ARBE, TCA-element, AuxRR-core, P-box, and TGA-element) as well as stress-related elements (including ARE, MBS, LTR, and GC-motif). These findings suggest that *AoHsfs* are closely associated with hormonal regulation and stress responses. Similar *cis*-elements, such as *ABRE*, *TGA-element*, *AuxRR-core*, and P-box, have been identified in Hsf members from other plant species like *Dianthus caryophyllus* and *Hypericum perforatum*. This suggests that these elements are conserved across different plant species and likely play a significant role in regulating Hsf genes^50^*.*

Furthermore, certain *cis*-acting elements, including LTR, ARE, and MBS, were also identified in Hsf genes from other plant species like *Brassica juncea* and *Camellia sinensis*^48^. Similar *cis*-acting elements in the promoter regions of Hsf genes from different plant species suggest that these elements are characteristic features of *Hsfs* and play a role in their regulation. Additionally, identifying collinear genes, such as *AT1G32330* as a collinear gene of *AoHsf11*, suggests that *AoHsf11* may have a role in responding to abiotic stress. The fact that *AoHsfs* show more collinear relationships with soybean than with rice and Arabidopsis could be attributed to differences in genome size and gene duplication events within the Hsf family. Also, *GLYMA_02G278400*, *GLYMA_14G036200* and *GLYMA_03G175300* had a connection with stresses through genome-wide association study as the collinearity gene of *AoHSF*s^51,52^.

Seeding is a critical phase in a plant's life cycle, as it significantly influences plant development and ultimately affects yield. Moreover, this stage is susceptible to various abiotic stresses, which can profoundly impact plant growth and productivity^53^. Identifying the target tissue for understanding the function of *AoHsfs* is crucial in unraveling their roles in plant growth and stress responses^54^. In this study, the roots have been chosen as the primary target tissues for further investigation due to their consistently elevated expression levels of *AoHsfs* both during the seeding stage and in response to abiotic stress conditions*.* Similarly, in the case of *PvHsfs*, the root also emerged as the most suitable tissue for studying their functions and roles due to their significant expression levels in this organ^46^. The expression levels of *AoHsfs* significantly changed under abiotic stresses, indicating their involvement in responding to these stresses. However, it's worth noting that not all Hsf members necessarily respond to every type of abiotic stress. Therefore, it is crucial to identify broad-spectrum genes that can react to multiple abiotic stresses, providing a more comprehensive defense mechanism for the plant^46^. HSF members had an important role under heat stress in lilies (*Lilium longiflorum*), which HSF members were considered as differentially expressed genes while HSF-HSP pathway was considered as a candidate pathways; *PsnHSF21* improved salt tolerance by specifically binding to the stress-related *cis*-acting element HSE while the transgenic poplar overexpressing plant had a better growth state under salt stress. .

The study demonstrates that the expression levels of many *AoHsf* members changed under abiotic stress conditions, suggesting their potential roles in responding to such stress. Members like *AoHsf05*, *AoHsf13*, and *AoHsf15* exhibited significantly higher expression levels under all three tested abiotic stresses, which these three members all had.hormone and stress related *cis*-acting elements (Fig. 5). This finding suggests that these particular *AoHsfs* could be promising candidates for breeding programs to enhance stress tolerance in asparagus.

## Conclusions

In this study, we identified 18 *AoHsfs* through the reference genome. Subsequently, we conducted a comprehensive analysis to gain insights into the evolutionary relationships, motifs, gene structures, collinearity, and expression patterns of these *AoHsfs*. Our findings revealed that these *AoHsfs* could be classified into five distinct clades, each showing similarities in motifs, gene structure, and *cis*-acting elements. Moreover, due to their higher expression levels, we identified roots as a suitable target tissue for studying AoHsfs during the seeding stage. Additionally, AoHsf05, AoHsf13, and AoHsf15 exhibited significant upregulation under various abiotic stress conditions, making them promising candidates for enhancing stress tolerance through breeding programs. This study provides valuable insights into the characterization of *AoHsfs* and highlights potential candidates for future breeding efforts.

### Supplementary Information

Supplementary Table S1.

Supplementary Table S2.

Supplementary Table S3.

Supplementary Table S4.

Supplementary Table S5.

## Data Availability

*Datasets*: The datasets analyzed during this study could be available from the corresponding author on reasonable request.
