# Supplementary material for: Genome-wide identification and comprehensive analysis heat shock transcription factor (Hsf) members in asparagus (Asparagus officinalis) at the seeding stage under abiotic stresses
Source: Sci Rep. 2023 Oct 23;13:18103. doi: 10.1038/s41598-023-45322-w (PMC10593832; doi:10.1038/s41598-023-45322-w)
Supplement: Supplementary file 1 — Supplementary Table S1. [file 41598_2023_45322_MOESM1_ESM.docx]

Table S1 The primers of AoHsf members in qRT-PCR analysis.

| No. | Forward Primer | Reverse Primer |
| --- | --- | --- |
| AoActin | CCAAGGCAGAGTACGATGAA | CCACCTCAAGACAGCTAGATAC |
| AoHsf01 | CCCAAAAACAAATAAAAA | TGAGAGAAAGCAAAAGGA |
| AoHsf02 | AAATCCTCAACCTCCGTA | TCCTCTTCTTTCCACTGC |
| AoHsf03 | CTACTGTTGTTCTCACCG | AATCTTAGGACTTTCTGC |
| AoHsf04 | TCCTCCTTCGGGTTCCTT | TCATCTTCGCCTGCCTCT |
| AoHsf05 | TACCTCTCGGCTCTCTCG | CGGCTCCTCCATTATTGT |
| AoHsf06 | TAAACTACACCCACAAAT | CTCAACTCAAATAACACA |
| AoHsf07 | TCAATACCTATGGTTTCA | CAACTTCAACACACGCTG |
| AoHsf08 | CTTCTCCAGTTTTGTTCG | TCCTCTTCATCGTCTTCG |
| AoHsf09 | CTGGAATCGTAAAGAAAC | CTCAACCAAGAAAGACAT |
| AoHsf10 | CAGATAAGAGAGAGCAGG | TTAGCACAATAACAAAGA |
| AoHsf11 | TGATAGAAGAGCAGAATG | GAATAAAACGCAAAAGAT |
| AoHsf12 | ATATATCCTTCCGTCACA | AAATTAAATCAATCAGCC |
| AoHsf13 | TGGCTTCCGCAAGGTGG | CGTGGCAGTGTTGGTGTT |
| AoHsf14 | ATGATAAAATGGAGGACC | TTCAACTTTGAAGGACGT |
| AoHsf15 | TCGTCACTACAGACAAAA | GTGAGACCATTAGACAGT |
| AoHsf16 | GATAGATGTTGGGTGCTC | ATCCGCTTCAAAGTTAAG |
| AoHsf17 | TGTTGCCTCGGTATTTCA | AGCATCTTTTCCCTTTCT |
| AoHsf18 | CTGACCGATGGGAGTTTG | GGCAGTGAGTATGGCTTT |
