# Supplementary material for: Genome-wide identification and comprehensive analysis heat shock transcription factor (Hsf) members in asparagus (Asparagus officinalis) at the seeding stage under abiotic stresses
Source: Sci Rep. 2023 Oct 23;13:18103. doi: 10.1038/s41598-023-45322-w (PMC10593832; doi:10.1038/s41598-023-45322-w)
Supplement: Supplementary file 2 — Supplementary Table S2. [file 41598_2023_45322_MOESM2_ESM.docx]

Table S2: The detailed information of AoHsf members.

| Gene name | Gene Bank | Gene_id | chr | Location | | CDS length | Protein length | Instability index | Aliphatic index |
| --- | --- | --- | --- | --- | --- | --- | --- | --- | --- |
| AoHsf01 | XP_020244690.1 | gene-LOC109822839 | NC_033794.1 | 36723462 | 36725312 | 1764 | 481 | 57.04 | 72.72 |
| AoHsf02 | XP_020261510.1 | gene-LOC109837603 | NC_033794.1 | 95049372 | 95050981 | 1640 | 504 | 42.04 | 69.96 |
| AoHsf03 | XP_020273532.1 | gene-LOC109848423 | NC_033794.1 | 129462641 | 129469510 | 1459 | 307 | 44.44 | 69.54 |
| AoHsf04 | XP_020254188.1 | gene-LOC109831266 | NC_033795.1 | 65697694 | 65700635 | 1011 | 230 | 45.25 | 80.96 |
| AoHsf05 | XP_020254681.1 | gene-LOC109831708 | NC_033795.1 | 78070789 | 78072557 | 1968 | 395 | 61.19 | 77.01 |
| AoHsf06 | XP_020259400.1 | gene-LOC109835786 | NC_033796.1 | 112334151 | 112343217 | 646 | 170 | 67.62 | 65.35 |
| AoHsf07 | XP_020262491.1 | gene-LOC109838458 | NC_033797.1 | 32099673 | 32105248 | 1881 | 284 | 56.64 | 54.61 |
| AoHsf08 | XP_020268219.1 | gene-LOC109843678 | NC_033798.1 | 21634579 | 21635975 | 1614 | 410 | 66.26 | 64.66 |
| AoHsf09 | XP_020276091.1 | gene-LOC109850490 | NC_033800.1 | 59339152 | 59340666 | 1656 | 467 | 53.89 | 64.99 |
| AoHsf10 | XP_020273360.1 | gene-LOC109848328 | NC_033800.1 | 135948234 | 135966759 | 1040 | 251 | 43.78 | 75.66 |
| AoHsf11 | XP_020242275.1 | gene-LOC109820529 | NC_033801.1 | 1848877 | 1852460 | 3937 | 384 | 55.9 | 80.39 |
| AoHsf12 | XP_020277134.1 | gene-LOC109851420 | NC_033801.1 | 68038940 | 68053729 | 1005 | 288 | 56.19 | 74.44 |
| AoHsf13 | XP_020244153.1 | gene-LOC109822373 | NC_033801.1 | 90151441 | 90156473 | 1338 | 434 | 59.71 | 71.29 |
| AoHsf14 | XP_020243291.1 | gene-LOC109821518 | NC_033801.1 | 91638058 | 91644272 | 1000 | 264 | 53.47 | 78.56 |
| AoHsf15 | XP_020248909.1 | gene-LOC109826301 | NC_033803.1 | 25212585 | 25214545 | 1209 | 256 | 65.42 | 65.51 |
| AoHsf16 | XP_020247414.1 | gene-LOC109825098 | NC_033803.1 | 60682249 | 60691306 | 1640 | 365 | 63.89 | 72.88 |
| AoHsf17 | XP_020248654.1 | gene-LOC109826099 | NC_033803.1 | 67594192 | 67595682 | 991 | 253 | 58.9 | 74.7 |
| AoHsf18 | XP_020250060.1 | gene-LOC109827465 | NW_017972489.1 | 191289 | 199779 | 1944 | 309 | 52.2 | 73.2 |
