# Supplementary material for: Genome-wide identification and comprehensive analysis heat shock transcription factor (Hsf) members in asparagus (Asparagus officinalis) at the seeding stage under abiotic stresses
Source: Sci Rep. 2023 Oct 23;13:18103. doi: 10.1038/s41598-023-45322-w (PMC10593832; doi:10.1038/s41598-023-45322-w)
Supplement: Supplementary file 4 — Supplementary Table S4. [file 41598_2023_45322_MOESM4_ESM.docx]

Table S4 The motifs information of Hsf members

| No. | The sequence of motifs |
| --- | --- |
| Motif 1 | 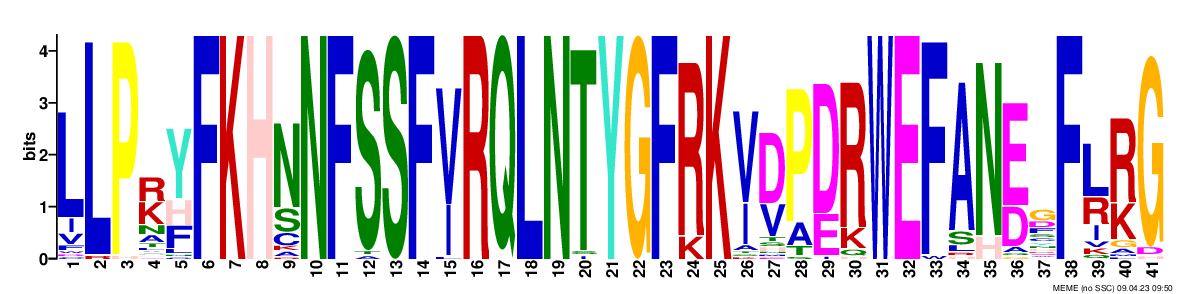 |
| Motif 2 | 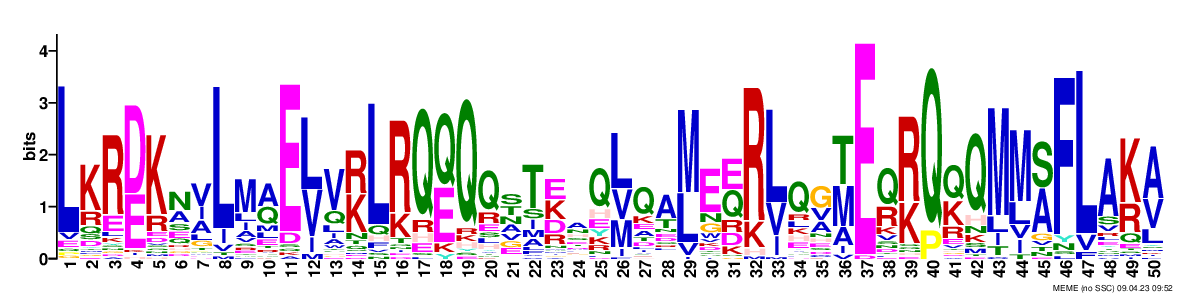 |
| Motif 3 | 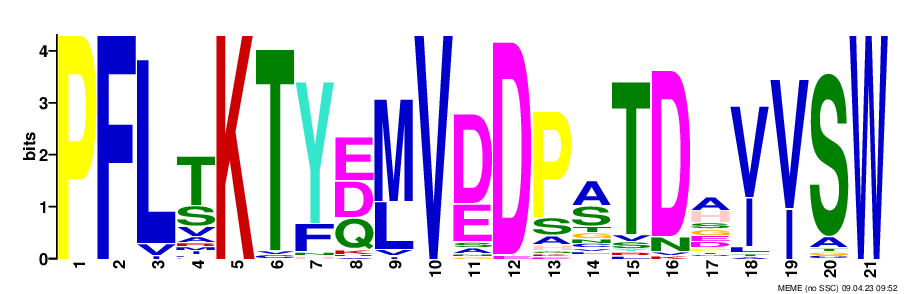 |
| Motif 4 | 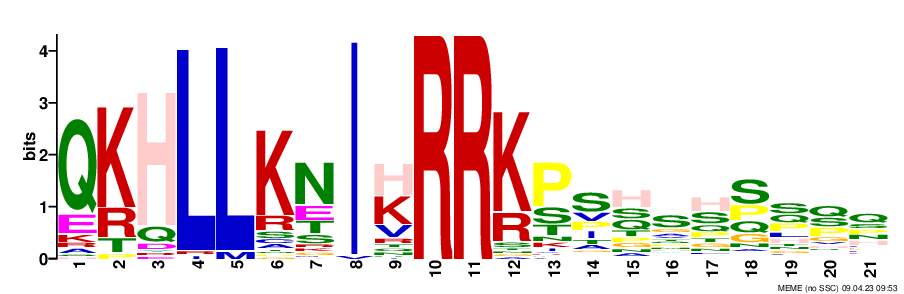 |
| Motif 5 | 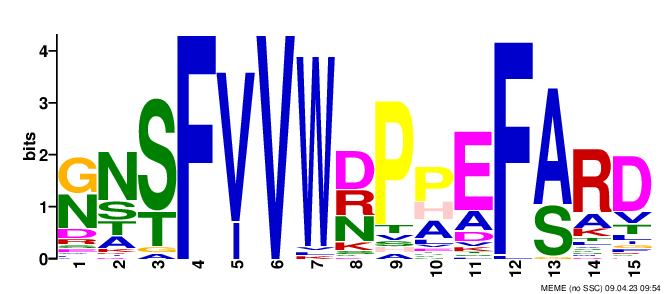 |
| Motif 6 | 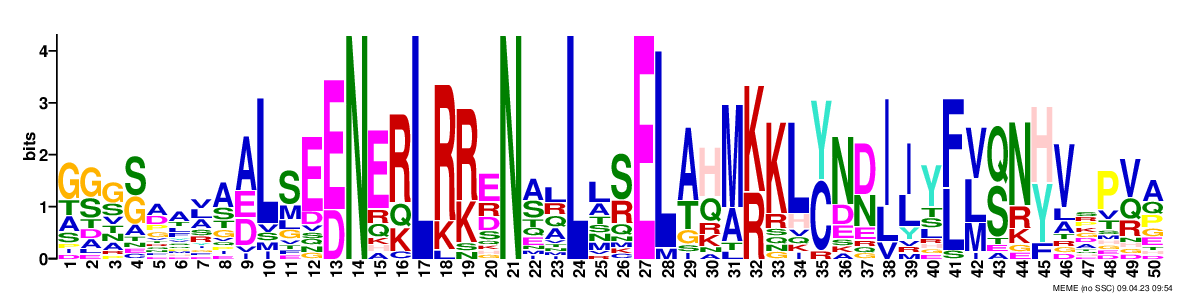 |
| Motif 7 | 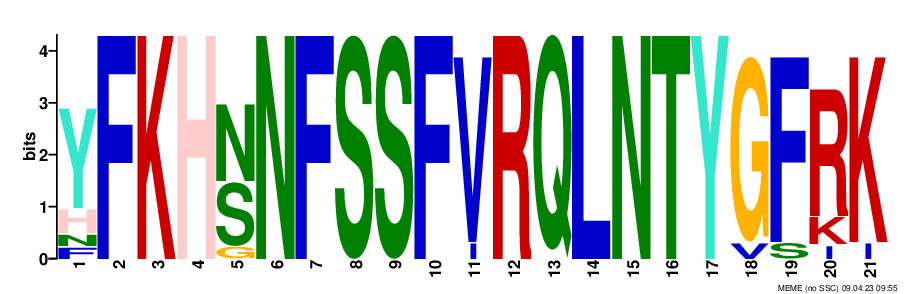 |
| Motif 8 | 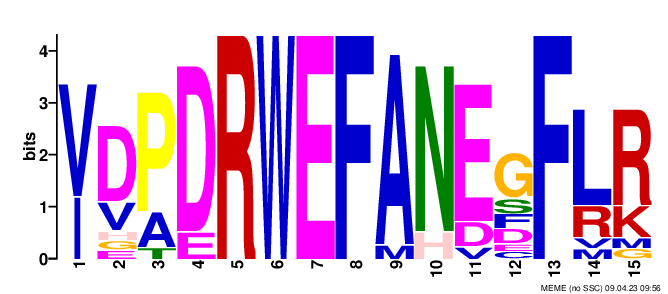 |
| Motif 9 | 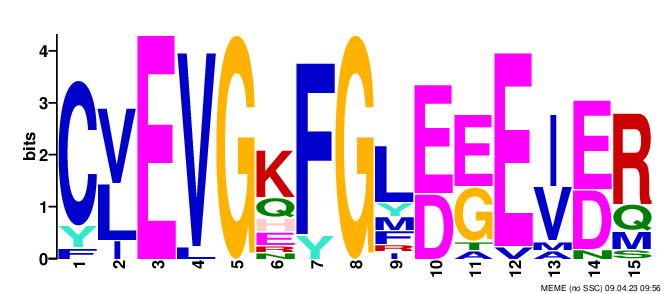 |
| Motif 10 | 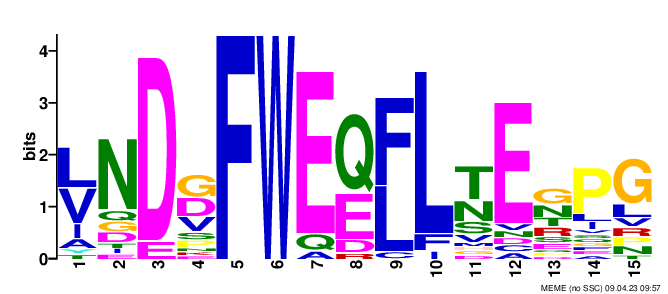 |
| Motif 11 | 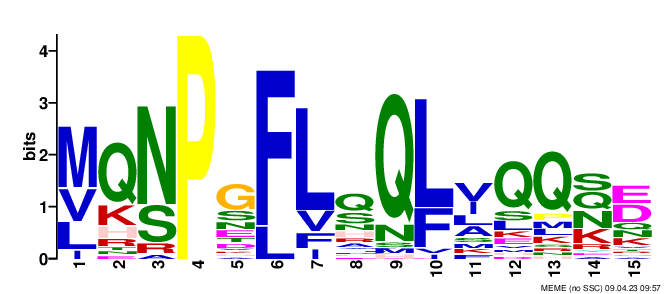 |
| Motif 12 | 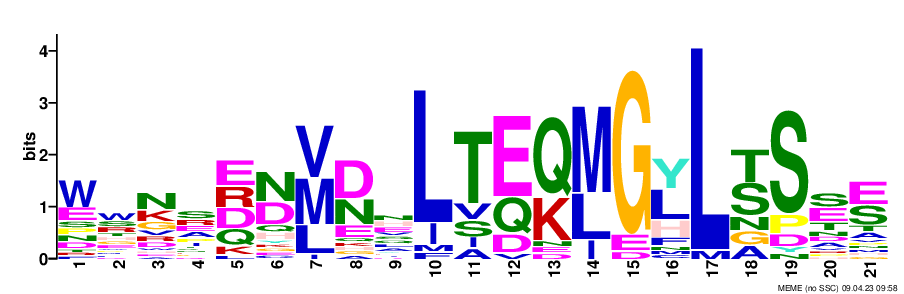 |
| Motif 13 | 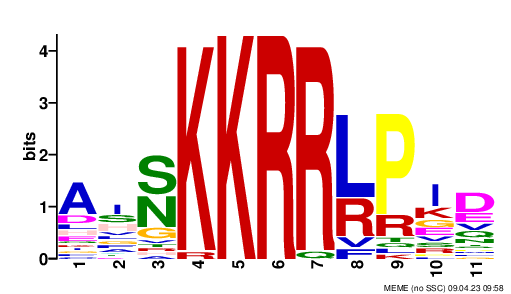 |
| Motif 14 | 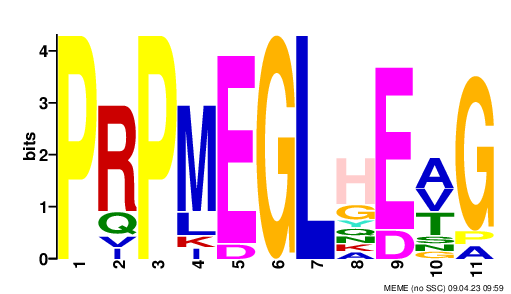 |
| Motif 15 | 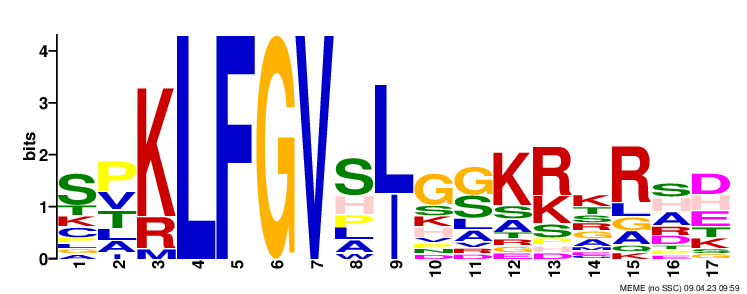 |
| Motif 16 | 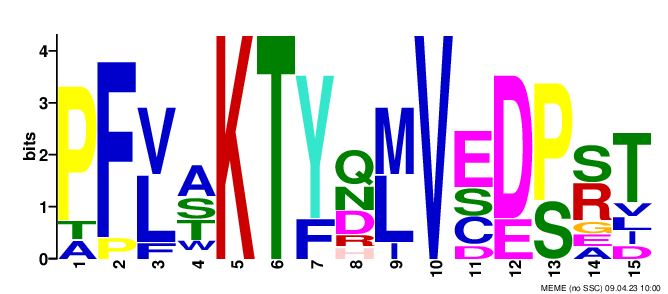 |
| Motif 17 | 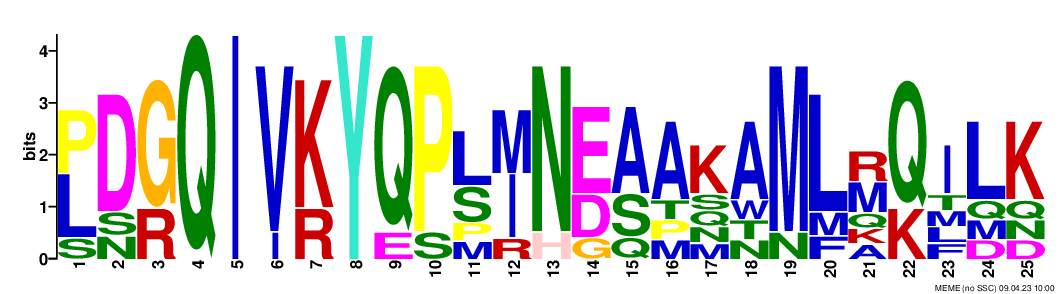 |
| Motif 18 | 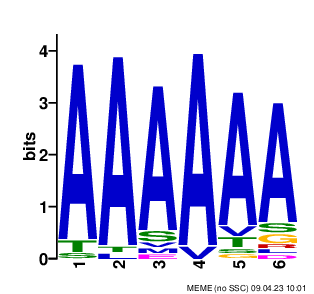 |
| Motif 19 | 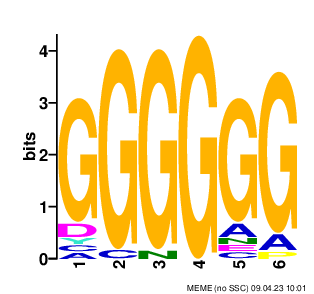 |
| Motif 20 | 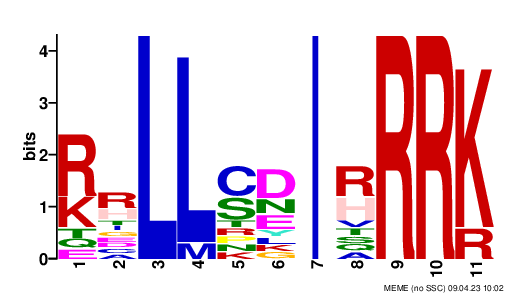 |
