# Supplementary material for: Genome-wide identification and comprehensive analysis heat shock transcription factor (Hsf) members in asparagus (Asparagus officinalis) at the seeding stage under abiotic stresses
Source: Sci Rep. 2023 Oct 23;13:18103. doi: 10.1038/s41598-023-45322-w (PMC10593832; doi:10.1038/s41598-023-45322-w)
Supplement: Supplementary file 5 — Supplementary Table S5. [file 41598_2023_45322_MOESM5_ESM.docx]

Table S5 The collinearity of genes and annotation

| Gene | Collinear genes | Species | `Annotation |
| --- | --- | --- | --- |
| *AoHsf05* | *GLYMA_14G036200* | *Glycine max* | PTHR10015//PTHR10015:SF177-HEAT SHOCK TRANSCRIPTION FACTOR |
| *AoHsf05* | *GLYMA_02G278400* | *Glycine max* | PTHR10015//PTHR10015:SF177-HEAT SHOCK TRANSCRIPTION FACTOR |
| *AoHsf10* | *GLYMA_03G157300* | *Glycine max* | PTHR10015//PTHR10015:SF160-HEAT SHOCK TRANSCRIPTION FACTOR |
| *AoHsf11* | *AT1G32330* | *Arabidopsis* | PTHR10015//PTHR10015:SF162-HEAT SHOCK TRANSCRIPTION FACTOR // HEAT STRESS TRANSCRIPTION FACTOR A-1A-RELATED |
| *AoHsf13* | *Os09t0456800* | *Oryza sativa* | PTHR10015//PTHR10015:SF166-HEAT SHOCK TRANSCRIPTION FACTOR // HEAT STRESS TRANSCRIPTION FACTOR B-1 |
| *AoHsf14* | *Os09t0455200* | *Oryza sativa* | PTHR10015//PTHR10015:SF204-HEAT SHOCK TRANSCRIPTION FACTOR // SUBFAMILY NOT NAMED |
